# Salvage hypospadias repairs

**DOI:** 10.4103/0971-9261.44763

**Published:** 2008

**Authors:** V. Sripathi, M. Satheesh, K. Shubha

**Affiliations:** Sundaram Children's Hospital and Apollo Hospitals, Chennai, India

**Keywords:** Buccal mucosal grafts, hypospadias, salvage repairs, snodgrass repair

## Abstract

**Aim:**

Review of our experience and to develop an algorithm for salvage procedures in the management of hypospadias cripples and treatment of urethral strictures following hypospadias repair.

**Methods:**

This is a retrospective review of hypospadias surgeries over a 41-month period. Out of a total 168 surgeries, 20 were salvage/re-operative repairs. In three children a Duplay repair was feasible, while in four others a variety of single-stage repairs could be done. The repair was staged in seven children – buccal mucosal grafts (BMGs) in five, buccal mucosal tube in one, and skin graft in one. Five children with dense strictures were managed by dorsal BMG inlay grafting in one, vascularized tunical onlay grafting on the ventrum in one, and a free tunical patch in one. Three children were treated by internal urethrotomy and stenting for four weeks with a poor outcome.

**Results:**

The age of children ranged from 1.5–15 years (mean 4.5). Follow-up ranged from 3 months to 3.5 years. Excellent results were obtained in 10 children (50%) with a well-surfaced erect penis and a slit-like meatus. Glans closure could not be achieved and meatus was coronal in three. Two children developed fistulae following a Duplay repair and following a staged BMG. Three repairs failed completely – a composite repair broke down, a BMG tube stenosed with a proximal leak, and a stricture recurred with loss of a ventral free tunical graft.

**Conclusions:**

In salvage procedures performed on hypospadias cripples, a staged repair with buccal mucosa as an inlay in the first stage followed by tubularization 4–6 months later provides good results. A simple algorithm to plan corrective surgery in failed hypospadias cases and obtain satisfactory results is devised.

## INTRODUCTION

To get good results consistently in hypospadias surgery, case load of 40 cases a year is considered essential.[1] However, redo surgery or surgery on cripples is an unique challenge. This is because there is often extensive scarring and lack of skin, deficiency of the glans from repeated attempts at closure, residual chordee, and meatal stenosis. A distinct wariness and suspicion on the part of the parents completes the picture.

At the very first visit, the surgeon needs to be honest about what can be reliably achieved and the time frame in which it can be done. If a staged repair is the only option, especially with tissue transfer from other areas, a detailed discussion with the parents is mandatory. With meticulous planning salvage surgery is very gratifying. However, there is always a possibility that transferred tissue may fail to take. In such cases, patients may sink into deep despair and may completely turn away from all forms of surgical treatment.

## PATIENTS AND METHODS

This is a retrospective review of the experience of a single surgeon (VS) over a 41-month period (January 2005 to May 2008). During this period, 168 hypospadias surgeries were done, of which, 128 were primary repairs, 20 were fistulae closures, and 20 were classified as salvage/reoperative repairs. These were children in whom there was complete breakdown of the repair, significant residual chordee, dense strictures or large fistulae (especially distal ones) where the repair had to be taken down completely and redone.

The children ranged in age from 1.5–15 years with a mean of 4.5 years. Of the 20 cases four were our own, while 16 were referrals from other centers. Prior to the salvage attempt, 13 had undergone one surgery while seven had undergone two or more attempts at repair. The average number of anesthetic procedures including those for dilatation was 3.5 per child. The orifice position was distal in eight, mid penile in six, and proximal penile in six. Eight children had significant residual chordee. The primary repair was a Mathieu flip-flap repair in five cases, an onlay graft in four cases (pedicled, 3; and double faced, 1), a pedicled tube (Duckett) in four cases, and Thiersch-Duplay tubularization in two cases. A Denis Browne buried-strip repair, a Koff (glans approximation repair), and a composite repair (tubularization and onlay) was done in one case each. One repair could not be categorized either by perusal of records or by visual assessment.

Before surgery the need for hormonal therapy was assessed. Four of the children were given at least one injection of testosterone enanthate (2 mg/kg) one month before salvage to increase shaft length and to increase vascularity of the available skin.

Our surgical strategy after degloving the penis and chordee correction was as follows:

1. If the urethral plate is unscarred and wide, tubularized incised plate (TIP) was our first choice (six cases).
2. If the plate was not available but there was redundant skin, Duplay tubularization (three cases), double-faced onlay (one case), or a composite repair (part tubularization and part onlay – one case) was done.
3. If the penis was short of skin and extensively scarred, urethral substitution was chosen and staged buccal mucosal grafts (BMGs) were our first choice (six cases). In one case, a local penile skin graft was used.

Buccal mucosal harvesting was done preferably from the inner cheek after identifying and avoiding the Stensen's duct or from the lower lip. The grafts were then placed on the penile defect and meticulously quilted in place with 6/0 or 7/0 PDS sutures. The graft take was assessed after 10 days. During this period, bladder was drained by a silastic Foley catheter. The child was reviewed on a monthly basis to monitor graft take and contraction. Second-stage tubularization was done six months later. At this time if the graft showed focal scars or narrowing, a second buccal mucosal or skin inlay was done before tubularization.

Five children had severe strictures – two in midshaft, two penoscrotal, and one distal. In two of them, direct vision internal urethrotomy (DVIU) was attempted and urethra stented with a silastic Foley catheter for four weeks. Definitive treatment of the stricture was done by buccal mucosal inlay on the dorsal aspect (one case), vascularized tunical patch on the ventral aspect (one case), and free tunical patch on the ventral aspect (one case). In one child, a perineal urethrostomy was established and the child was given a testosterone injection to increase penile size prior to staged BMG. All children were commenced on oral dexamethasone in an attempt to prevent progression of the stricture.

## RESULTS

Our follow-up ranged from 3–36 months. Criteria for a successful repair included a straight penis, glans closure, meatus at the tip and ‘slit-like’, and adequate resurfacing of the penile shaft. These could be achieved in ten children (50%). In three children, glans closure could not be achieved due to glans scarring/deficiency and the meatus was placed in the corona. In two children, fistulae were noted one year following a Thiersch-Duplay repair and three months following a buccal mucosal graft. In one child, 30 months following a TIP repair the urinary stream was still thin though there was no postvoid residue on ultrasound and uroflow was reasonable. In one child, shaft skin was deficient and though closure was achieved, the result was felt to be suboptimal.

In three children the salvage repair failed. In one child, a composite repair broke down completely and the parents opted out of follow-up when a two-stage repair was suggested. In the second child, a buccal mucosal tube stenosed resulting in a leak from the perineal anastomosis. Dilatation and catheterization was done in an attempt to salvage the repair. In the third child, there was necrosis of a tunica vaginalis free graft placed on the ventral aspect of a stricture and a dorsal buccal mucosal inlay was planned.

In a 10-year-old with a mid-urethral stricture, DVIU and stenting was done to temporize the situation following urinary extravazation. On follow-up, the uroflow was poor (8 ml/sec) on a voided volume of 150 ml and there was a large postvoid residue. However, further surgery was postponed.

## DISCUSSION

Following meticulous review of our results, we propose an algorithm for the operative management of reoperative/salvage hypospadias [Tables 1 and 2] as follows:

**Table 1:** Algorithm for redo repairs when urethral plate is present

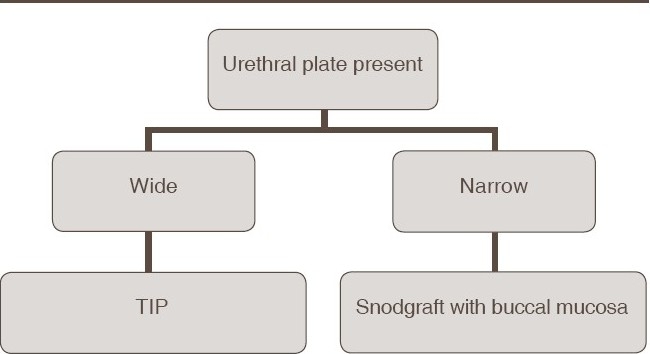

**Table 2:** Algorithm for redo repairs when urethral plate is absent or scarred

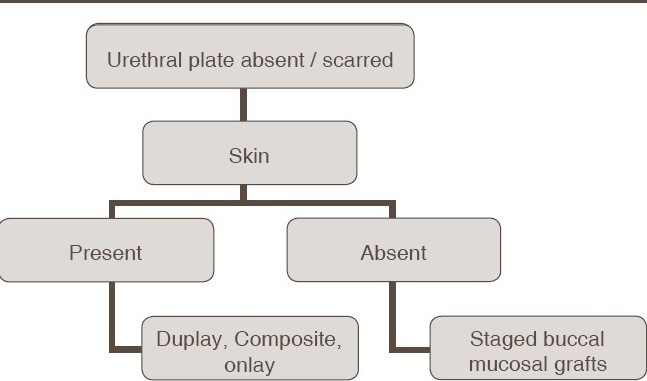

### (a) Assessment of meatus and need for testosterone therapy

At the first visit the urethral meatus is assessed. If the meatus is stenotic a meatotomy with a generous cut-back to normal tissue is done. Thereafter, an assessment is done for the need for testosterone therapy. Though normally advised for primary repairs it is nevertheless useful for redo's also, if penile length can be gained and vascularity of overlying skin improved prior to surgery. Preoperative testosterone cream application is used routinely by the Mainz group.[2] As testosterone cream is not available in our country, we used injectable testosterone enanthate in four of our cases.

### (b) Urethral plate intact – TIP and Snodgraft

**Figure 1:**
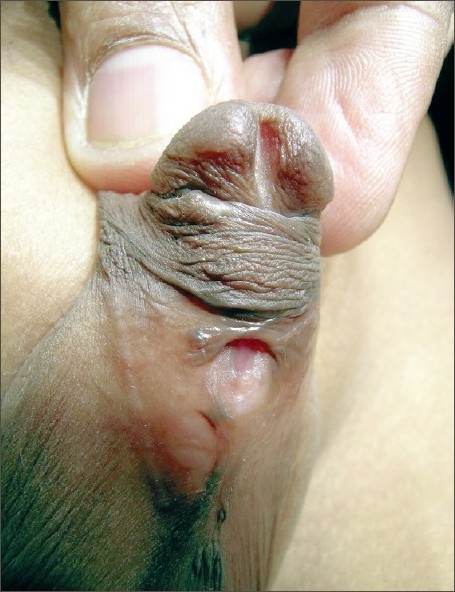
Complete breakdown of an onlay flap repair showing intact urethral plate – suitable for tubularized incised plate (TIP) repair

**Figure 2:**
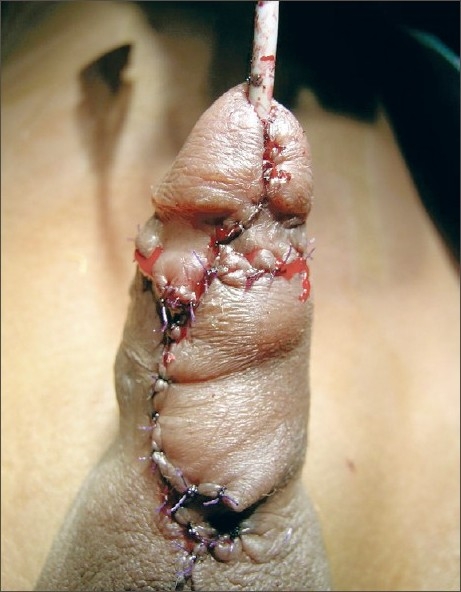
Completed TIP repair of penis shown in figure 1

When the urethral plate is intact, unscarred, and wide, tubularized incised plate urethroplasty (TIP) is a logical choice [Figures 1 and 2]. When outlining the urethral plate one may ‘borrow’ skin on either side to enable an easy closure around an 8-Fr stent. We employed the TIP repair in six cases with excellent results. In the follow-up period if the parents complained of a thin stream or a prolonged voiding time, we did not hesitate to calibrate the urethra under anesthesia. In all children, uroflow study six months to one year postoperation and an ultrasound to assess postvoid residue was mandatory. In one child, two sittings of dilatation were deemed necessary and urine stream was still thin, 2.5 years later. However, uroflow has remained normal and repeated ultrasound exams have shown no postvoid residue.

When the urethral plate is narrow we would choose to enlarge it by a midline incision and laying a BMG to increase its diameter (inlay graft). This has been reported as a point of technique by Hayes and Malone in 1999 and christened as augmented Snodgrass or ‘Snodgraft’ by Bracka.[3,1] This technique enables a single-stage closure and is largely trouble free. When this option is kept at the back of mind then the problems of tubularizing narrow urethral plates and keeping small stents are largely eliminated. This, in our opinion, is the chief cause of troublesome stenosis and complaints of a thin stream. In our series, the child who had a persistently narrow stream following a TIP on review was found to have been tubularized around a 6-Fr stent. In Yang's excellent review of reoperatives, Snodgrass, stress has been rightly laid on the width of the urethral plate and the size of the stent.[4] The stent should be two sizes smaller than the urethral width. For example, in children less than six years the recommended width of the urethral plate is 12 mm with an 8-Fr stent. In a 14-year old, the width of the separated urethral plate should be 18 mm and the stent 14 Fr. To avoid strictures following TIP, Snodgrass in 1999 declared that the minimum width of the urethral plate after incision should be 10 mm.[5] Coupled with Elder's criteria on meatal sizes at various ages, choosing an appropriately sized stent should not be difficult.[4] In one of our cases, the urethral plate was reincised after a previous TIP. At reincision the plate was supple and the repair was uneventful. Though this has found wide acceptance Yang *et al*, caution that postoperative fistula rates in reincision cases is high.[4]

### (c) Poor urethral plate – plenty of skin

If the urethral plate is deemed unsuitable for TIP, the availability of skin would determine our choice of repair. In three cases, a Duplay tubularization could be done. In all these instances, we have closed the skin by a ‘double breasting’ technique. In one case, the dorsal skin was relatively undisturbed and we could get a good result with a double-faced onlay graft on a narrow urethral plate. In one case the urethra was tubulated to mid shaft and the repair completed with an onlay graft from the dorsal skin.

### (d) Poor urethral plate – no skin – staged buccal mucosal grafts

**Figure 3:**
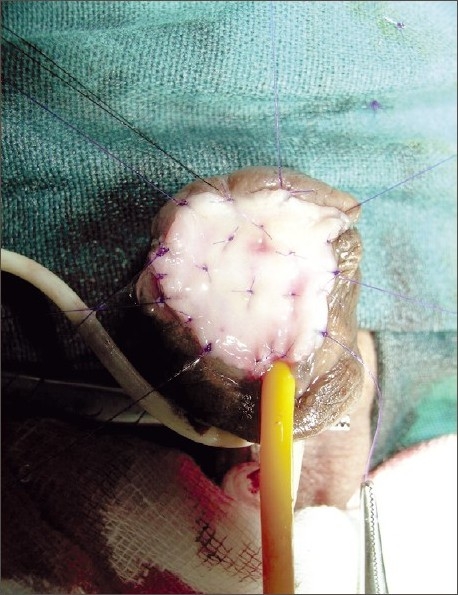
Scar tissue excised, glans laid open, and buccal mucosal graft (BMG) quilted in place

When the urethral plate is missing or is scarred, our preferred technique of substitution urethroplasty is a two-stage closure using buccal mucosa.[1,6] This tissue is easy to harvest and is robust. It has a thin submucosa allowing easy inosculation and a thick epithelial layer. We have employed this in three instances with excellent results [Figure 3]. One child developed a fistula in the mid shaft but is voiding well from the tip and awaiting closure.

In a five-year review of buccal mucosal onlay graft repairs Fechner *et al*, claim that problems will usually declare themselves in the first year after the repair.[2] This article stresses on the need to oversize the graft by 20% to allow shrinkage before tubularization six months later. As suggested by Snodgrass, we review our BMGs every month after the first stage.[6] Any areas of focal scarring or necrosis are regrafted by a buccal mucosal inlay prior to tubularization. In a review of 62 salvage hypospadias repairs using buccal mucosa, the Mainz group has indicated that it may not be desirable to bring the meatus to the the tip by doing a glansplasty.[7] They believe that this induces an area of high pressure at the tip which may encourage blowouts proximally. In three children in our series, glans scarring precluded closure and the meatus was kept at the corona. Important guidelines in the use of staged BMGs from various publications have been summarized for easy review in Table 3.

**Table 3:** Useful points to remember when undertaking buccal mucosal grafts for reoperative hypospadias

Grafts should be oversized by 20% to account for shrinkage
Meticulous ‘defatting’ is required for good take
Lower lip grafts being thinner are preferred for the glans and meatal area
Grafts should be securely quilted in position both in the center and at the edges
Compression ‘tie over’ dressing for 10 days is essential for good graft take
Monthly review is useful to assess focal scars which can be regrafted prior to tubularization
Tubularization can be done in 4–6 months time. Grafts can be left open to air without undue effects
Cheek donor areas can be closed primarily
Lower lip donor area should be left open
Dairy products should be avoided for 72 hours to speed-up healing

**Figure 4:**
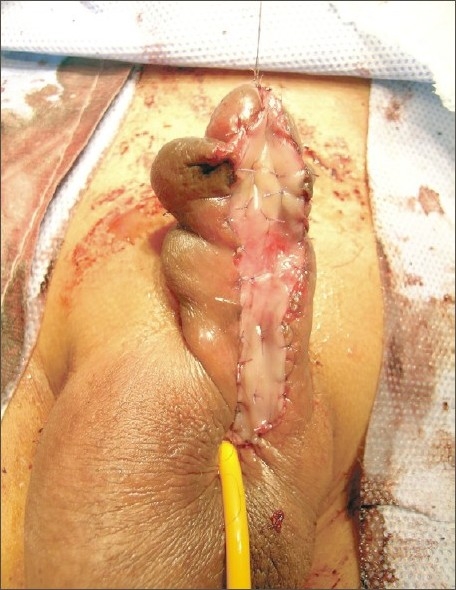
Long buccal mucosal grafts of >5 cm laid side-to-side and quilted. Note inlay into dorsal urethra

Our single case of a buccal mucosal tube has been a failure due to stenosis. The results have been uniformly disappointing with tubes – 50% complication as opposed to 20% with onlay patches.[8] In the same article it was noted that a patch longer than 5 cm in length was likely to give rise to problems. We noted that in one of our cases when two long grafts were laid side-by-side one of the grafts was lost with a recurrence of meatal stenosis [Figure 4]. In retrospect, we believe that a buccal mucosal onlay at the first sitting would have served our purpose with probably a better result.

### (e) Urethral strictures

Direct vision internal urethrotomy and stenting of urethral strictures was done in three cases with poor results. In a review comparing DVIU with DVIU and stenting, Hussman *et al*, have observed uniformly poor results in both the groups. This has been attributed to extravasation of fluid, urine, and blood into the periurethral tissues following DVIU. Due to the lack of a spongiosal layer, this extravasation is not contained and results in a significant inflammatory response which further reduces urethral blood supply and causes stricture progression.[9]

For the definitive treatment of strictures, the laying of buccal mucosa on the dorsal aspect of the urethra on the backbone of cavernosal tissue has found to yield excellent results following the report of Barbagli *et al*.[10] We have adopted a modification in which the strictured area was approached through a ventral sagittal urethrotomy and the buccal mucosal inlay was placed on the dorsal aspect after stricture division and quilted in position. Excellent results have been reported in 12 adults using this technique.[11] A ventral approach and widening of the strictured area with a tunical patch on a vascular pedicle has also given excellent results in one case. However, in a 13-year old a ventral approach and patching of the stricture with a free tunical graft did not work even though a vascularized covering layer was provided.

## References

[1] Manzoni G, Bracka A, Palminteri E, Marrocco G (2004). Hypospadias surgery: When, what and by whom?. BJU Int.

[2] Fichtner J, Filipas D, Fisch M, Hohenfellner R, Thuroff JW (2004). Long-term follow-up of buccal mucosal onlay graft for hypospadias repair: Analysis of complications. J Urol.

[3] Hayes MC, Malone PS (1999). The use of a dorsal buccal mucosal graft with urethral plate incision (Snodgrass) for hypospadias salvage. BJU Int.

[4] Yang SS, Chen SC, Hsieh Ch, Chen YT (2001). Reoperative snodgrass procedure. J Urol.

[5] Snodgrass W (1999). Does tubularised incised plate hypospadias repairs create neourethral strictures?. J Urol.

[6] Snodgrass W, Elmore J (2004). Initial experience with staged buccal graft (Bracka) hypospadias reoperations. J Urol.

[7] Fichtner J, Fisch M, Filipas D, Thuroff JW, Hohenfellner R (1998). Refinements in buccal mucosal graft urethroplasty for hypospadias repair. World J Urol.

[8] Hensle TW, Kearney MC, Bingham JB (2002). Buccal mucosal grafts for hypospadias surgery: Long term results. J Urol.

[9] Husmann DA, Rathbun SR (2006). Long-term follow up of visual internal urethrotomy for management of short (less than 1 cm) penile urethral strictures following hypospadias repair. J Urol.

[10] Barbagli G, Selli C, di Cello V, Mottola A (1996). A one-stage dorsal free-graft urethroplasty for bulbar urethral strictures. Br J Urol.

[11] Gupta NP, Ansari MS, Dogra PN, Tandon S (2004). Dorsal buccal mucosal graft urethroplasty by a ventral sagittal urethrotomy and minimal-access perineal approach for anterior urethral stricture. BJU Int.

